# Validation of the International Consultation on Incontinence Questionnaire-Pediatric Lower Urinary Tract Symptoms (ICIQ-CLUTS) for Spanish-speaking children

**DOI:** 10.1007/s00431-023-04823-6

**Published:** 2023-01-19

**Authors:** Maria Blanco-Diaz, Alvaro Manuel Rodriguez-Rodriguez, Jose Casaña, Sergio Hernandez-Sanchez

**Affiliations:** 1 Faculty of Medicine and Health Sciences, Physiotherapy and Translational Research Group (FINTRA-RG), Institute of Health Research of the Principality of Asturias (ISPA), University of Oviedo, Oviedo, 33006 Spain; 2 Exercise Intervention for Health Research Group (EXINH-RG), Department of Physiotherapy, University of Valencia, Valencia, 46010 Spain; 3 Department of Pathology and Surgery, Physiotherapy Area, Center for Translational Research in Physiotherapy, Miguel Hernandez University, San Juan de Alicante, Elche, 03550 Spain

**Keywords:** Questionnaire validation, Urinary incontinence, Urination disorders, Paedriatic, Urinary tract

## Abstract

The International Consultation on Incontinence Questionnaire-Pediatric Lower Urinary Tract Symptoms (ICIQ-CLUTS) is a 12-item self-administered tool to screen lower urinary tract symptoms (LUTS) in children. The aim of this study is to translate and validate the ICIQ-CLUTS into Spanish (ICIQ-CLUTS-Sp) and to study its psychometric properties. The cross-cultural adaptation of the ICIQ-CLUTS was performed following international recommendations. The psychometric analysis of the ICIQ-CLUTS-Sp was carried out to determine the reliability, validity, and diagnostic accuracy in a sample of 155 children and parents who completed the Spanish version ICIQ-CLUTS. The reliability indicators for the ICIQ-CLUTS-Sp were excellent (Cronbach’s alpha was > 0.8 and ICC > 0.9 both for children’s and parents’ versions). There was a high Pearson *r* > 0.6 and a high agreement level between children’s and parents’ answers (ICC > 0.6), except in 4 items. For parents, the standard error of measurement (SEm) was 0.41, and the minimal detectable change (MDC) was 1.14 points. In children, these results were 0.42 and 1.16 points, respectively. Cut-off points greater than 15 points in the parent version or 16 points in the children version have the highest sensitivity and specificity for detecting LUTS.

*  Conclusion*: The Spanish version of the ICIQ-CLUTS questionnaire is a valid, reliable, and diagnostically accurate instrument to identify cases of children with LUTS. Therefore, it can be used to screen for lower urinary tract symptoms in Spanish speaking children and/or parents, as well as to monitor the effects of interventions.

**Table:** 

**What is Known:**
• *Lower urinary tract symptoms in children should be assessed multimodally using minimally invasive diagnostic procedures. One way to do this is to use the questionnaire to differentiate these cases in paediatric patients.*
• *A cross-cultural adaptation of the ICIQ-CLUTS questionnaire to Spanish has not yet been done.*
**What is New:**
• *Based on a comprehensive validation methodology, this study highlights that the ICIQ-CLUTSSp questionnaire has good psychometric properties.*

**Supplementary Information:**

The online version contains supplementary material available at 10.1007/s00431-023-04823-6.

## Introduction

Lower urinary tract symptoms (LUTS) apply to the disorder that occurs in any of the stages of urination due to anatomical and/or functional changes in the organs responsible for this process[1]. Its symptoms affect the lives of children and their caregivers, resulting in low self-esteem, social isolation, and behavioral changes including learning difficulties [2, 3].

The International Children’s Continence Society (ICCS) has reinforced the need for standardized terminology for symptoms of lower urinary tract dysfunction and advises on the use of robust assessment instruments to measure the effects of clinical interventions [4, 5]. LUTS in children should be evaluated in a multimodal way by minimal invasive diagnostic procedures [6].

For this purpose, the use of patient-centered instruments to assess symptoms of the lower urinary tract has increased mainly when it comes to pediatric urology [7]. In 2010, De Gennaro et al. [8] and the International Consultation on Incontinence Questionnaire Committee published the ICIQ-CLUTS questionnaire to differentiate cases of LUTS in children. It was originally published in English, German, and Italian. It consists of 12 items and has two versions, one for parents and another one for children aged 5–18 years [8].

However, to the best of the authors’ knowledge, a cross-cultural adaptation of the ICIQ-CLUTS questionnaire into Spanish has not yet been performed. Therefore, the aim of this study was to translate and cross-culturally adapt the ICIQ-LUTS into Spanish and to study its psychometric properties of reliability and validity.

## Materials and methods

A cross-cultural adaptation of the “ICIQ-CLUTS” questionnaire was carried out following the COSMIN recommendations [9].

The authors of the original study were contacted by email (personal communication with Dr. Gennaro), and their authorisation was obtained to carry out this study. The study protocol was approved by the Principality of Asturias Research Ethics Committee (Reference PA/22–16).

### Translation and cross-cultural adaptation

**Fig. 1:**
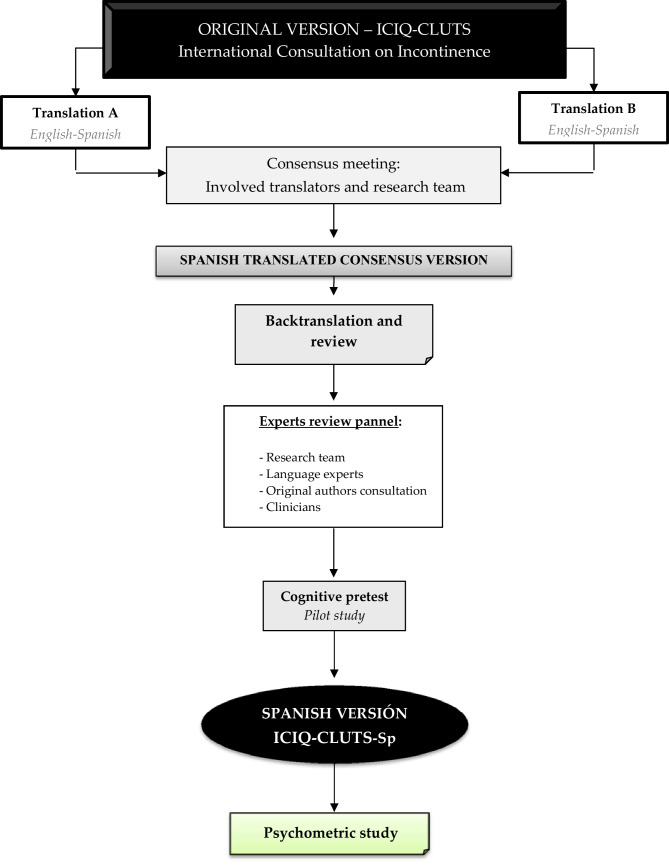
Translation and backtranslation ICIQ-CLUTS

The translation and backtranslation were performed as per international recommendations following the six-step process described by Beaton et al. [10]. Figure 1 shows a summary of the steps developed from the translation to the psychometric study.

In the first stage, the original English version was translated into Spanish by two independent translators. A unified version was obtained from the consensus. This version was backtranslated into English in a second stage by an independent translator who was not familiar with the original version.

In a third stage, the cross-cultural equivalence of the English and Spanish versions obtained in the previous stages was verified. The consensus version was submitted to a cognitive pre-test (fourth stage). Fifteen children and parents participated in this stage. Pre-test participants were asked to point out any ambiguities in the reading or answering style.

Finally, a psychometric study using the Spanish consensus version was carried out. Questionnaire, population, and procedures used in the validation phase are detailed below.

### Participants

A convenience sample of 155 children aged between 5 and 14 years, living in the Principality of Asturias (a province in the north of Spain), was recruited between January 2018 and March 2020. Health professionals (paediatricians and paediatric nurses) from hospitals and primary care centres in this region were contacted to recruit the study participants. Also, all the parents who consulted professionals for symptoms of incontinence in their children were also invited to participate. As in the original version, the following exclusion criteria were stablished: postoperative urological controls, patients with uncontrolled insulin dependent diabetes, and patients with anatomical abnormalities or neurological disorders were excluded from the study.

### Outcome measure−original questionnaire

This instrument is composed by 12 items. It is organized with special regard to age groups, including 5 to 9 (children), 10 to 13 (prepubertal patients), and 14 to 18 years old (postpubertal patients). The final score of the test ranges from 9 to 36. Responses for questions 4 to 12 (both included) are on a Likert-type scale from 1 to 4 points. Higher scores on the test indicate greater severity of LUTS in the child.

### Psychometric study

The following measurement properties of the ICIQ-CLUTS-Sp were analyzed: reliability (internal consistency and test–retest reliability), validity (construct validity and discriminant validity), and the floor and ceiling effects.

Reliability was assessed in terms of internal consistency of the items and for temporal stability (reproducibility). To assess the stability of responses to the questionnaire over time, the questionnaire was administered a second time to a sub-sample of 41 children, 2 weeks after the first assessment. Measurement error was also assessed by calculating the standard error of measurement (SEm) and minimum detectable change (MDC). The agreement between the children and parents scores for each ICIQ-CLUTS-Sp item and for the total score was evaluated.

Construct validity was assessed by studying the factor structure using an exploratory approach. For discriminant validity, a diagnostic accuracy analysis was performed, using the medical diagnosis of LUTS as a reference for positive cases. From the results of the receiver-operating characteristic (ROC) curves, the likelihood ratio (LR) was estimated as the ratio between the likelihood of observing a result in patients with this disease versus the likelihood of that result in patients without the pathology [12].

Ceiling and floor effects were also studied, considering to be present if more than 15% of the responders achieved the theoretical minimum or maximum possible score.

### Statistics

Descriptive statistics were used to describe the characteristics of the participants; mean and standard deviation were used for the quantitative variables and frequencies and percentages for the qualitative variables. For testing the normal distribution of the variables, the Kolmogorov–Smirnov test was used.

For the reliability study, internal consistency was studied by the Cronbach’s α coefficient and temporal stability by intraclass correlation coefficient (ICC_1,2_). The standard error of measurement (SEm) was estimated using the following formula: SD x $\sqrt{(1-R)}$, where SD is the standard deviation of the first assessment, and *R* is the reliability coefficient for the questionnaire [13]. The MDC threshold was calculated as 1.96 × $\surd 2$  × SEm.

The agreement level between the answers of the children and parents’ versions was also evaluated. For each item of the ICIQ-CLUTS-Sp, the mean and standard deviation for the children and parents’ versions were calculated, and the mean absolute difference (children minus parent value) was determined, including the effect sizes [14]. In addition, the Pearson coefficient and the ICC were calculated. The ICC type was a two-way random model (absolute agreement, average measures) (ICC_2,2_) [15]. For its interpretation, an ICC of 0.40 and below indicates poor agreement, 0.41–0.60 moderate agreement, 0.61–0.80 good agreement, and 0.81–1.00 excellent agreement [16].

Additionally, a Bland–Altman graphical representation was constructed by plotting the mean difference between the children and parents’ versions (ICIQ-CLUTS-Sp total score), with corresponding agreement limits (± 1.96SD) against their mean [17].

For exploratory factor analysis (EFA), principal component analysis was applied with Varimax rotation. For the factor extraction, the following conditions were considered: eigen value higher than 1.0 and accounting for more than 10% of variance [18, 19].

The Kaiser–Meyer–Olkin (KMO) measure of sampling adequacy was set at 0.7–1.0 to indicate adequate sampling, and the significance level of the Barlett test of sphericity was *p* < 0.001, indicating that the EFA could be used for the data analysis.

According to the authors of the original questionnaire, Gennaro et al., “Regarding to PCA, ICIQ-CLUTS has a multicomponent structure, which usually suggests that subscales/subscores could be more convenient from the psychometric viewpoint.” This implies that there are several dimensions or concepts that are assessed with the scale, and perhaps it would be convenient to study the internal structure in greater depth in the future by means of confirmatory factor analysis.

Internally, the scale includes aspects of urinary symptoms (urgency, voiding, etc.) and two items on infections and stool frequency, respectively. For practical purposes, there are no implications for administration or scoring.

A ROC curve was used to assess the diagnostic accuracy and to identify appropriate cutoff points and associated sensitivity and specificity values. Positive and negative likelihood ratios (LR) and area under the ROC curve (AUC) were also estimated. A LR + greater than 10 and a LR − lower than de 0.1 are considered to provide strong evidence to rule in or rule out diagnoses respectively in most circumstances [20, 21]*.* Considering the LRs and knowing the pretest probability (prevalence), the probability of detecting LUTS was calculated from a cut-off point on the ICIQ-CLUTS-Sp scale using the Fagan normogram [22].

The sample size calculation was estimated for the reliability study: for an alpha of 0.01, statistical power of 0.80, lower limit ρ_(0)_ = 0.7, upper limit ρ_(1)_ = 0.9, an expected ICC_2,1_ of 0.90, and 15% drop-out rate, and a total sample of 100 subjects was required..

Statistical analyses were conducted using the Statistical Package for Social Science version 24.0 (SPSS Inc., Chicago, Ill. USA) for Windows. The MedCalc software (MedCalc Software Ltd., Ostend, Belgium) was employed for the ROC curves calculation and plotting.

## Results

A sample of 155 children, aged between 5 and 14 years, as well as their respective parents, participated in the study. Table 1 shows the characteristics of the studied population. No missing responses were found in the data collection. ICIQ-CLUTS-Sp scores ranged from 11 to 27 points, with a mean of 17.2 ± 4.9 for parents. For children, the mean ICIQ-CLUTS-Sp was 17.1 ± 5.1 points (range 10 to 27).

**Table 1:** Descriptive characteristics of the studied population

	**5 to 9 years (*n*** **= 82)**	**10 to 14 years (*n*** **= 73)**
Age, years	7.3 ± 1.2	11.6 ± 1.6
Gender, *n* (%)		
Male	48 (58%)	40 (54%)
Female	34 (41%)	33 (45%)
LUTS diagnostic, *n* (%)	39 (47%)	34 (46%)
Baseline ICIQ-CLUTS-Sp		
Child	17.5 ± 5.1	16.7 ± 5.1
Parents	17.8 ± 5.1	16.6 ± 4.7
Retest ICIQ-CLUTS-Sp		
Child	19.0 ± 6.6	17.6 ± 7.4
Parents	19.4 ± 6.8	17.2 ± 6.7

### Reliability

Results from the reliability study and for the measurement error are presented in Table 2.

**Table 2:** Reliability indicators of the ICIQ-CLUTS-Sp scores

	**Cronbach alpha**	**ICC (95%CI)**	**SEm**	**MDC**
Children	0.82	0.993 (0.986 to 0.996)	0.42	1.16
5 to 9 years	0.81	0.994 (0.986 to 0.998)		
10 to 14 years	0.79	0.997 (0.993 to 0.999)		
Parents	0.81	0.994 (0.987 to 0.997)	0.41	1.14
5 to 9 years	0.82	0.992 (0.981 to 0.997)		
10 to 14 years	0.84	0.997 (0.992 to 0.999)		

*LUTS* lower urinary tract symptoms, *ICIQ-CLUTS-Sp* International Consultation on Incontinence Questionnaire-pediatric Lower Urinary Tract Symptoms Spanish version

### Agreement

The agreement indicators between the children’s answers and parents’ ones are presented in supplementary materials S1. The Bland–Altman plot (supplementary material S4) shows that most of the pairs differences in total ICIQ-CLUTS-Sp are between the agreement limits, which implies a high concordance between both versions in the ICIQ-CLUTS-Sp total score.

### Factor structure

The correlation matrix for the maximum likelihood extraction from the results observed in KMO values and the Bartlett’s test of sphericity were adequate. For parents, the KMO value was 0.81 with a significant Bartlett’s sphericity test result (*P* < 0.001). A factorial solution was obtained with 3 factors explaining 62% of the variance. The first factor would explain 39% of it, the second one would explain 13%, and the third factor would explain 10% of the total variance.

For children, the KMO value was 0.846 with a significant Bartlett’s sphericity test result (*P* < 0.001). A factorial solution was obtained with 2 factors explaining 56% of the variance. The first factor would explain 42% of the variance, and the second one 14% of the total variance.

Supplementary material S3 shows factor loadings for parents and children ICIQ-CLUTS-Sp versions respectively. A scree plot for children’s and parents’ versions is shown in supplementary material S5 and S6.

### Diagnostic accuracy

**Fig. 2:**
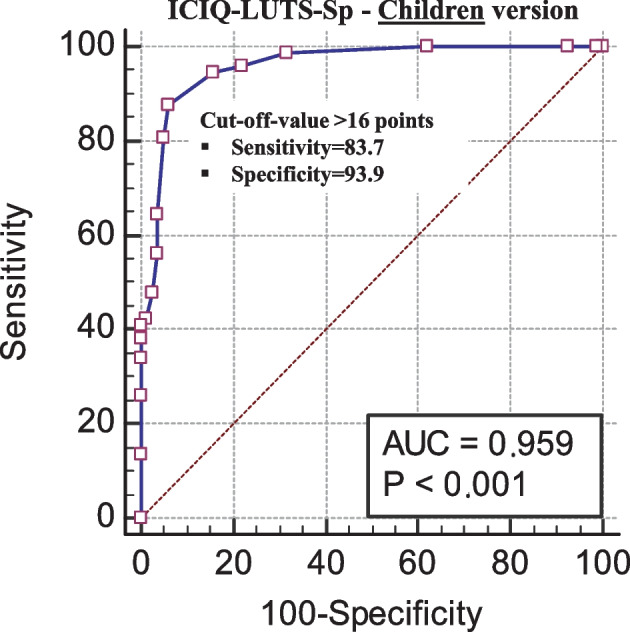
ROC curve children. Abbreviations: ICIQ-CLUTS-Sp, International Consultation on Incontinence Questionnaire-Pediatric Lower Urinary Tract Symptoms, Spanish version; AUC, area under the ROC curve

**Fig. 3:**
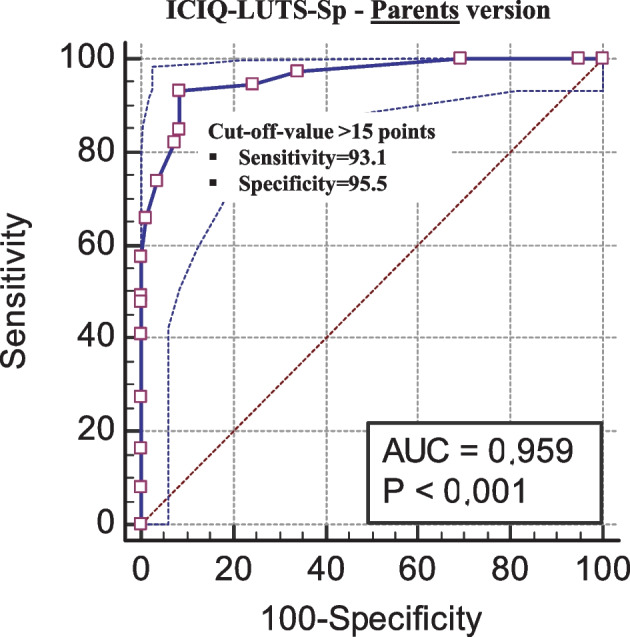
ROC curve parents. Abbreviations: ICIQ-CLUTS-Sp, International Consultation on Incontinence Questionnaire-Pediatric Lower Urinary Tract Symptoms, Spanish version; AUC, area under the ROC curve

A total score higher than 16 points on the ICIQ-CLUTS-Sp children’s version and 15 points on the parents’ version were identified as cut-off points discriminating children with LUTS from healthy children (Figs. 2 and 3). Table 3 shows the AUCs, sensitivity, and specificity values associated with these cutoffs, the likelihood ratios (positive and negative), and the probability posttest both for the children’s and the parents’ versions. All Fagan nomograms are included in Supplemental material 2.

**Table 3:** Results from the ROC curve analysis for ICIQ-CLUTS-Sp diagnostic accuracy

	**LUTS diagnostic**					**AUC 95%CI**						**Posttest probability**	
	**Positive**	**Negative**	**ROC cut-off point**	**AUC**	**SE**	**Lower bound**	**Upper bound**	**Sensitivity (95%CI)**	**Specificity (95%CI)**	**LR + (95%CI)**	**LR- (95%CI)**	**Positive**	**Negative**
Children	73	82	> 16	0.959	0.014	0.914	0.984	87.7 (77.9 to 94.2)	93.9 (86.3 to 98.0)	14.4 (6.1 to 33.8)	0.13 (0.07 to 0.2)	93%	10%
Parents			> 15	0.959	0.012	0.915	0.984	93.1 (84.7 to 97.7)	91.5 (83.2 to 96.5)	10.9 (5.4 to 22.2)	0.07 (0.03 to 0.2)	91%	6.3%
Subgroup 5 to 9 years (*n* = 82)													
Children	39	43	> 16	0.944	0.023	0.897	0.990	89.7 (75.8 to 91.2)	88.4 (74.9 to 96.1)	7.7 (3.4 to 17.7)	0.12 (0.05 to 0.3)	87%	10%
Parents			> 15	0.963	0.018	0.897	0.992	94.9 (82.7 to 99.4)	86.1 (72.1 to 94.7)	6.8 (3.2 to 14.3)	0.06 (0.02 to 0.2)	86%	5.2%
Subgroup 10 to 14 years (*n* = 73)													
Children	34	39	> 15	0.984	0.010	0.964	1.000	94.1 (80.3 to 99.3)	93.2 (79.1 to 98.4)	12.2 (4.1 to 36.4)	0.06 (0.02 to 0.2)	91%	5%
Parents			> 15	0.967	0.019	0.896	0.995	91.2 (76.3 to 98.1)	97.4 (86.5 to 99.9)	35.4 (5.1 to 246.1)	0.09 (0.03 to 0.3)	97%	7.1%

### Ceiling and floor effects

Any children nor parent achieved the highest or lowest possible score on the questionnaire. Therefore, no floor or ceiling effect was detected.

## Discussion

The International Consultation on Incontinence Questionnaire-Pediatric Lower Urinary Tract Symptoms (ICIQ-CLUTS) is a screening questionnaire for LUTS in children. To date, it is available in English, Italian, and German [8]. It was developed in child and parent self-administered versions. The ICIQ-LUTS has demonstrated good correlation with clinical impression and to be reliable and objective to grade LUTS in pediatric population [11]. It is a valid, reliable, and useful to screen for LUTS in pediatric population. A score of 14 points on the children version and 13 points on the parents version were identified as cutoff points discriminating children with LUTS from healthy children [8].

The aim of the present study was to make a cross-cultural adaptation of the ICIQ-CLUTS scale into Spanish for use in LUTS screening in children. The obtained version meets the appropriate criteria of reliability, validity, and diagnostic sensitivity.

According to literature, more than 10% of school children refer lower urinary tract dysfunction that requires specialised medical consultation [23]. It is a problem that generates great concern both for the children who suffer it and for their parents because of the physical, emotional, and social implications that it generates [24]. Therefore, the assessment of LUTS in children is a complex task that goes beyond organic dysfunction. Thus, the use of one or more instruments that collect the children’s and parents’ point of view on the lower urinary tract problem is recommended for a more global and complete assessment [5].

Different barriers to the use of Patient-Reported Outcome Measures (PROMs) in clinical care with the paediatric population have been identified in the literature [25]. In this case, the ICIQ-CLUTS questionnaire is feasible because it takes little time for children or parents to complete it (less than 4 min), and it is simple to score, reasons that facilitate its use in clinical daily routine.

There were no missing data. All the participants that were asked for participating, finally, they did it. All of them fulfilled the questionnaire, and this step was supervised by the doctor/nurse.

### Reliability

The reliability for the ICIQ-CLUTS-Sp scores were high and similar to those reported by the original version. In the analysis of internal consistency, there are no indicators pointing to an items’ redundancy, such as an Cronbach’s alpha threshold of 0.95 [26]. Indicators of temporal stability were very high which implies excellent retest reliability.

The measurement error indicators have not been previously calculated for ICIQ-CLUTS scale. The obtained SEm and MDC values are very low, demonstrating the scale’s capacity to detect real changes properly in the clinical situation as a consequence of changes of more than 2 points on the scale. In this sense, and continuing to improve its applicability, it would be appropriate to carry out future studies to determine the threshold of significant clinical change (MCID) of the scale [27].

Regarding the agreement in the answers given by children and their respective parents, there is a moderate-to-high agreement for all items, except for questions 3, 6, 9, and 12. In questions 6 and 12, which refer to urination and defecation frequency respectively, it is likely that the difference lies in the fact that parents during school hours or other play activities cannot count them exactly, and they could answer with an estimation. Children probably do not do this accurately either, but rather estimate the average for the day. In question 3, about urinary tract infections in the last month, it is striking that there is a low correlation. It may be because of children, even knowing the symptoms they have suffered, do not identify the entity as an infection. This could point to a review of the language used to ensure that children understand what they are being asked about.

Despite the high correlation of the scores of parents and children in general, the combination of the two versions is recommended as the most appropriate strategy for screening children with LUTS [28].

### Validity

The study of the factor structure of the ICIQ-CLUTS-Sp by means of principal component analysis has confirmed its similarity with the results obtained in the original work. Firstly, different structures are obtained for the parents’ and children’s version respectively. Also, in the parents’ version, even having two factors with only one item which could be considered as not very strong from the psychometric point of view [19], up to three factors are identified that meet the requirements established a priori, as happened in the original work. In the case of the children, we obtained a two-factor internal structure, which is not a three-factor structure but the second factor brings together three items (3, 6, and 9), which can be considered as strong, and the total variance explained is higher than the original study by De Gennaro et al. [8].

### Diagnostic accuracy

In the diagnostic accuracy study, all AUC curves indicated excellent ability of the ICIQ-LUTS-Sp to discriminate between children with and without LUTS. A scale of these characteristics will be more useful to the extent that its LR + is of greater magnitude, since it allows to confirm with greater certainty the presence of disease, and its LR − has a low value (Loong [12]). In this case, both positive (> 10) and negative (< 1) LR values confirm the high accuracy of the instrument for LUTS screening in children. Using the Fagan nomogram, it can be seen how, for example, in the parent version using the cut-off point of > 16 points on the questionnaire, there is a probability greater than 91% of having a positive diagnosis of LUTS while, with a score below this threshold, the probability of not having LUTS is 6.3%. This analysis gives the ICIQ-CLUTS-Sp scores a very applied character in clinical practice [22]. These cut-off points can be used in the clinical context as a simple and rapid screening.

Some limitations should be considered when interpreting the results of the present investigation. First, a subgroup of 14–18-year-old patients was not included as in the original study as this group is not considered in the paediatric segment in Spain. Second, it would have been desirable to use another scale with the same objective or a quality-of-life instrument in order to obtain evidence of convergent validity. However, the centre dynamics and the short duration of the visits made it very difficult to extend the evaluation dossier for feasibility reasons. Finally, regarding the factorial structure and considering the results obtained in our study, it is necessary to carry out future studies with larger samples in order to clarify the dimensions of the scale by means of a confirmatory factor analysis.

The original sample is 155 children aged 5–14 years, and both parents and children in the 5–9 age group were asked. Although the ratio is not significant for children versus parents, some children (especially 5–6 years old) may be just starting to learn how to read and may have difficulty understanding the questions of the questionnaire. This subgroup analysis has not been carried out, but it can be recommended that parents ask the questions to their children rather than the children answering the questions in the questionnaire directly.

The Spanish adaptation of the ICIQ-CLUTS scale showed no problems in translation. In the psychometric study, excellent indicators of reliability and diagnostic sensitivity were obtained, with cut-off points higher than 15 points in the parents’ version or 16 points in the children’s version, being those with the highest sensitivity and specificity for detecting LUTS.

With this new version of the ICIQ-CLUTS scale, paediatricians and urologists will be able to use it in Spanish-speaking children and parents to detect and quantify the magnitude of LUTS, as well as to monitor the effects of their interventions.

## Supplementary Information

Below is the link to the electronic supplementary material.

Supplementary file1 Agreement indicators for children´s and parents´ answers (TIFF 15087 KB)

Supplementary file2 Fagan Normograms (PPTX 370 KB)

Supplementary file3 Rotated component matrix with loadings for each extracted factor in the Parents (P) and Children (CH) versions (TIFF 15087 KB)

Supplementary file4 Bland Altman plot ICIQ-CLUTS-Sp total score (TIFF 30170 KB)

Supplementary file5 Scree plot children (TIFF 30170 KB)

Supplementary file6 Scree plot parents (TIFF 102 KB)

## Abbreviations

AUC: Area under the ROC curve
EFA: Exploratory factor analysis
ICC: Intraclass correlation coefficient
ICCS: International Children’s Continence Society
ICIQ-CLUTS: International Consultation on Incontinence Questionnaire-Pediatric Lower Urinary Tract Symptoms
ICIQ-CLUTS-Sp: Spanish International Consultation on Incontinence Questionnaire-Pediatric Lower Urinary Tract Symptoms
KMO: Kaiser-Meyer-Olkin
LR: Likelihood ratio
LUTS: Lower urinary tract symptoms
MCID: Significant clinically important difference
MDC: Minimal detectable change
PROMs: Patient-Reported Outcome Measures
ROC: Receiver-operating characteristic
SD: Standard deviation
SE: Standard error
SEm: Standard error of measurement
