# Supplementary material for: Validation of the International Consultation on Incontinence Questionnaire-Pediatric Lower Urinary Tract Symptoms (ICIQ-CLUTS) for Spanish-speaking children
Source: Eur J Pediatr. 2023 Jan 19;182(3):1361–9. doi: 10.1007/s00431-023-04823-6 (PMC10023609; doi:10.1007/s00431-023-04823-6)
Supplement: Supplementary file 2 — Supplementary file2 Fagan Normograms (PPTX 370 KB) [file 431_2023_4823_MOESM2_ESM.pptx]

## Slide 1
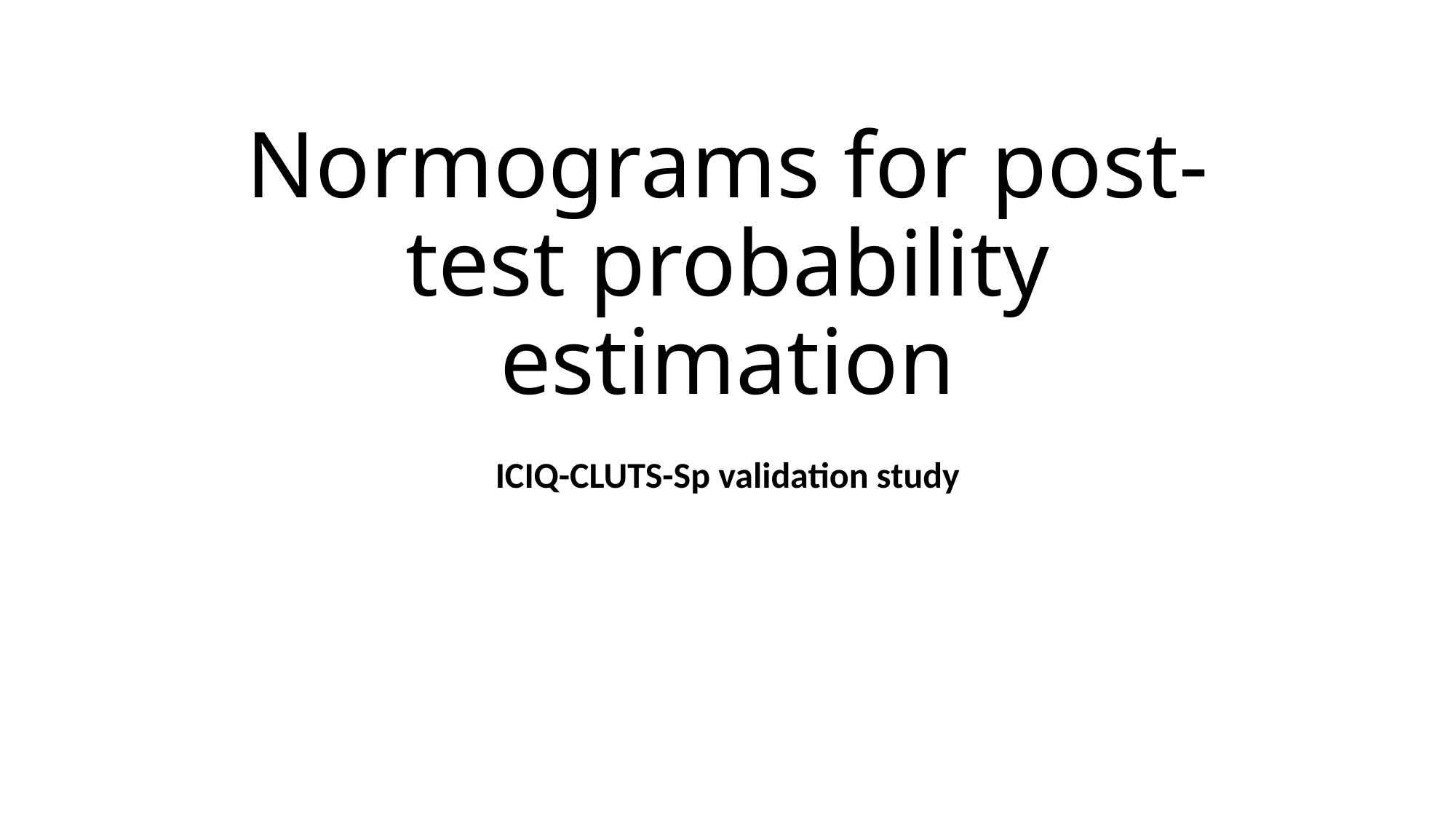

# Normograms for post-test probability estimation
ICIQ-CLUTS-Sp validation study

## Slide 2
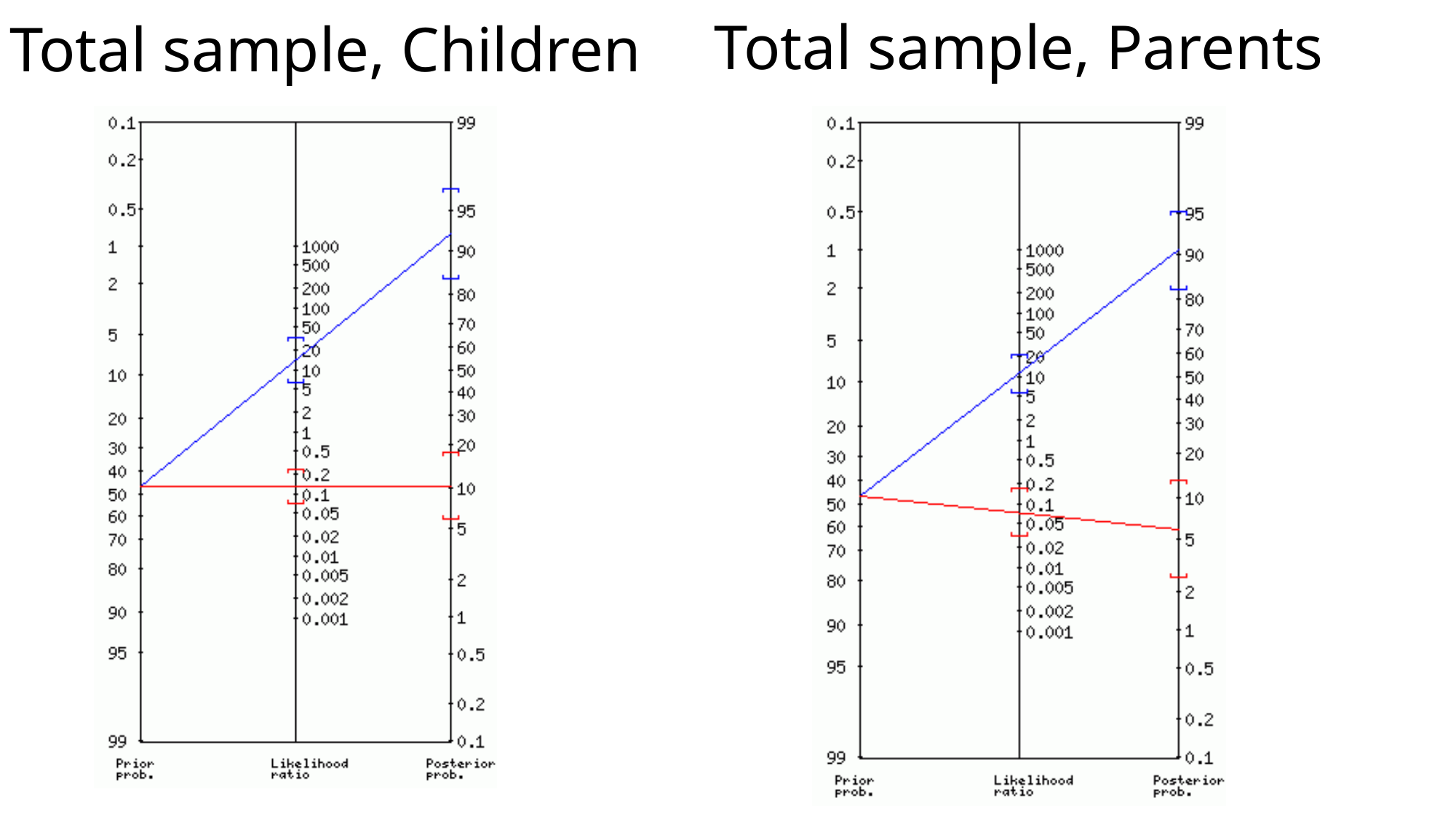

Total sample, Parents
Total sample, Children

## Slide 3
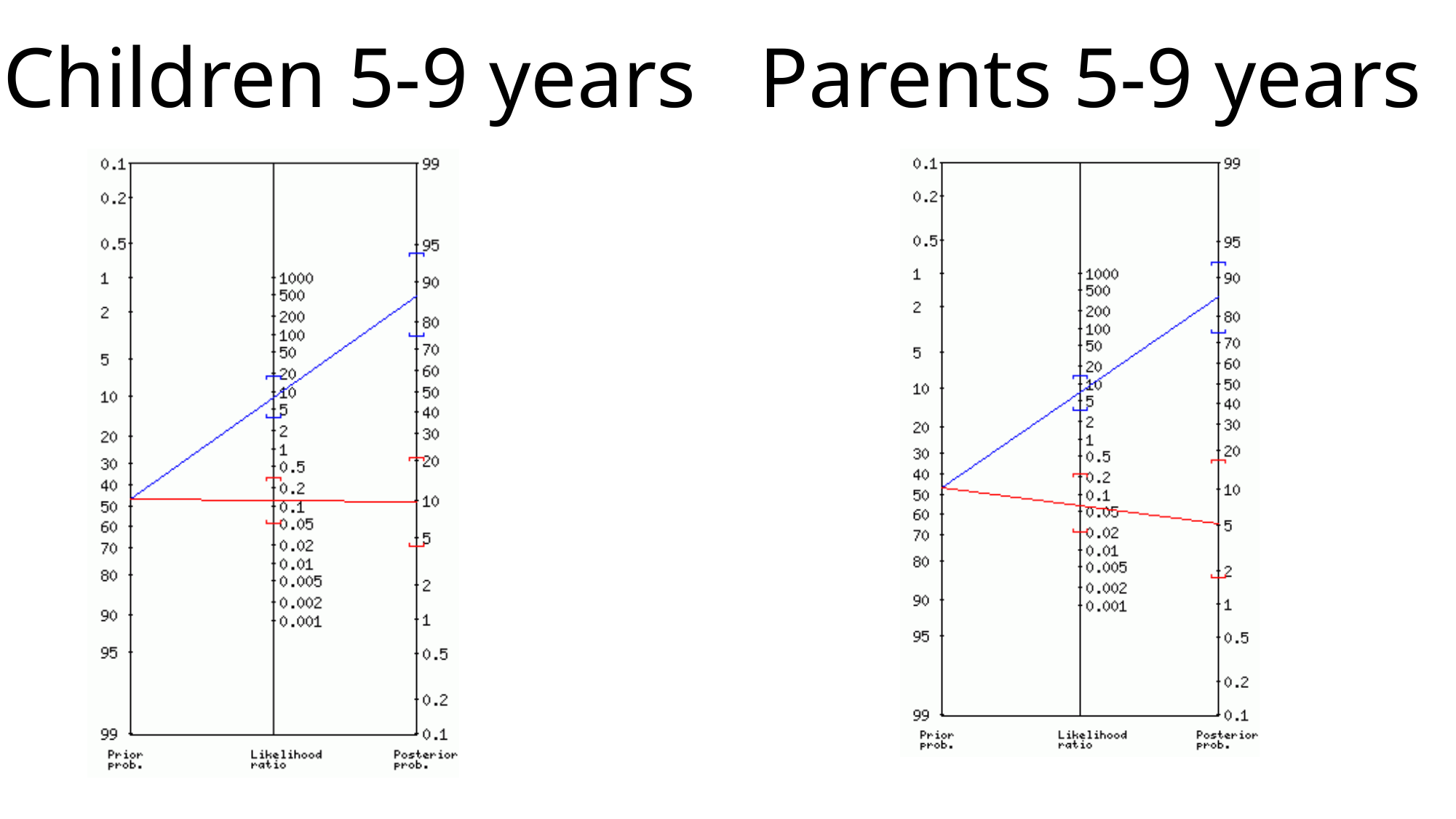

Children 5-9 years
Parents 5-9 years

## Slide 4
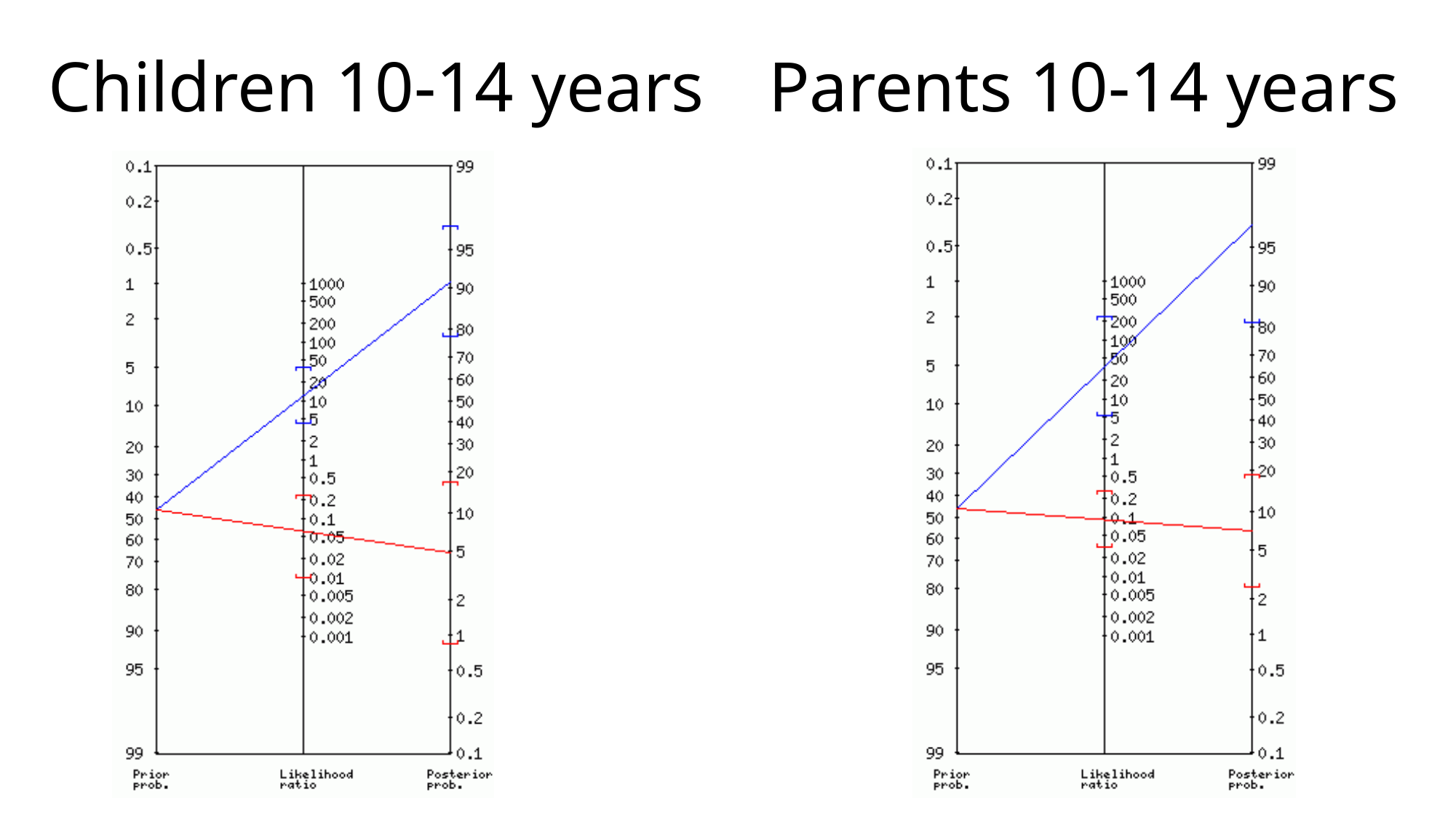

Children 10-14 years
Parents 10-14 years
