# Supplementary figures and images for: Validation of the International Consultation on Incontinence Questionnaire-Pediatric Lower Urinary Tract Symptoms (ICIQ-CLUTS) for Spanish-speaking children
Source: Eur J Pediatr. 2023 Jan 19;182(3):1361–9. doi: 10.1007/s00431-023-04823-6 (PMC10023609; doi:10.1007/s00431-023-04823-6)

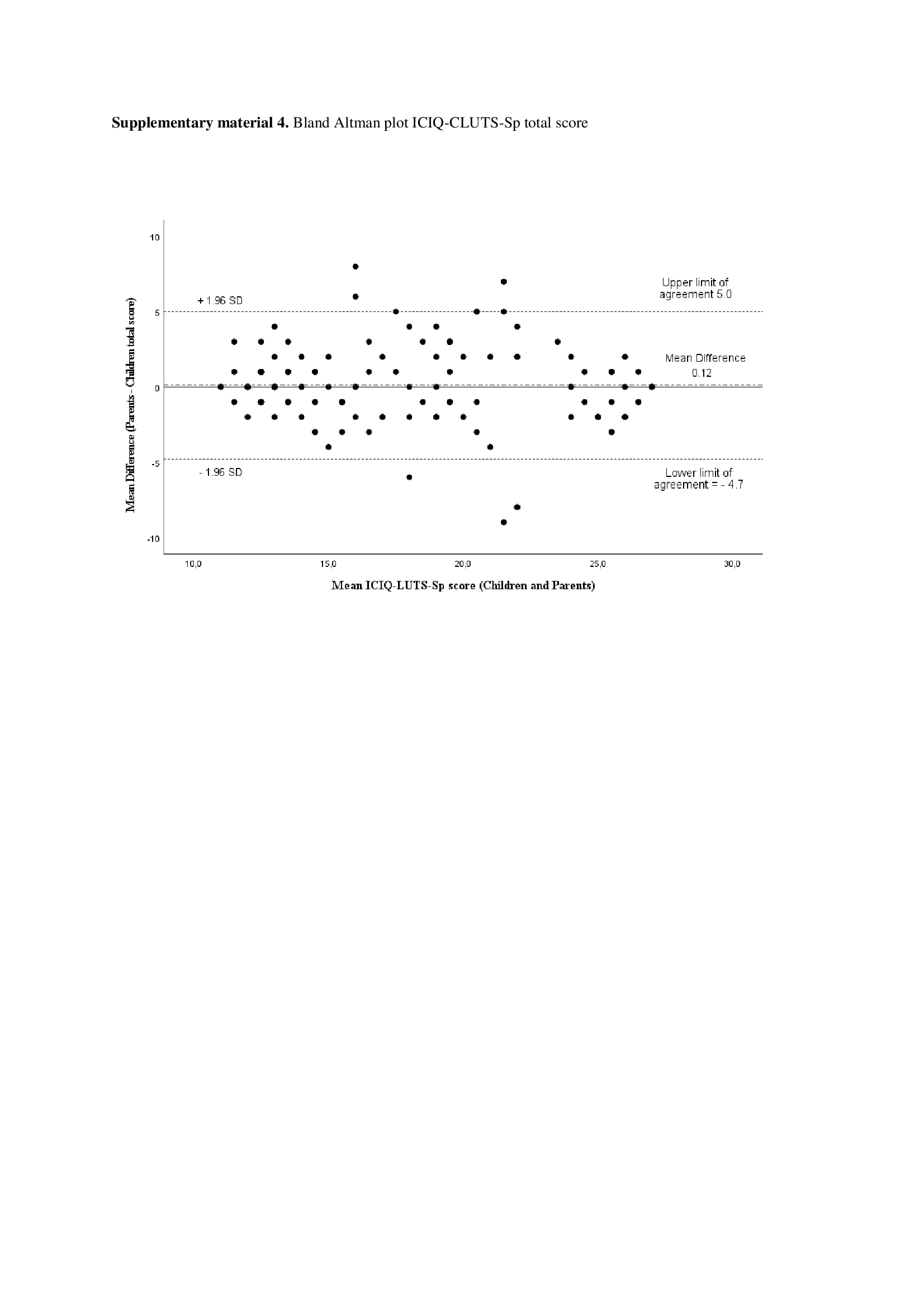

Supplement: Supplementary file 4 — Supplementary file4 Bland Altman plot ICIQ-CLUTS-Sp total score (TIFF 30170 KB) [file 431_2023_4823_MOESM4_ESM.tiff]

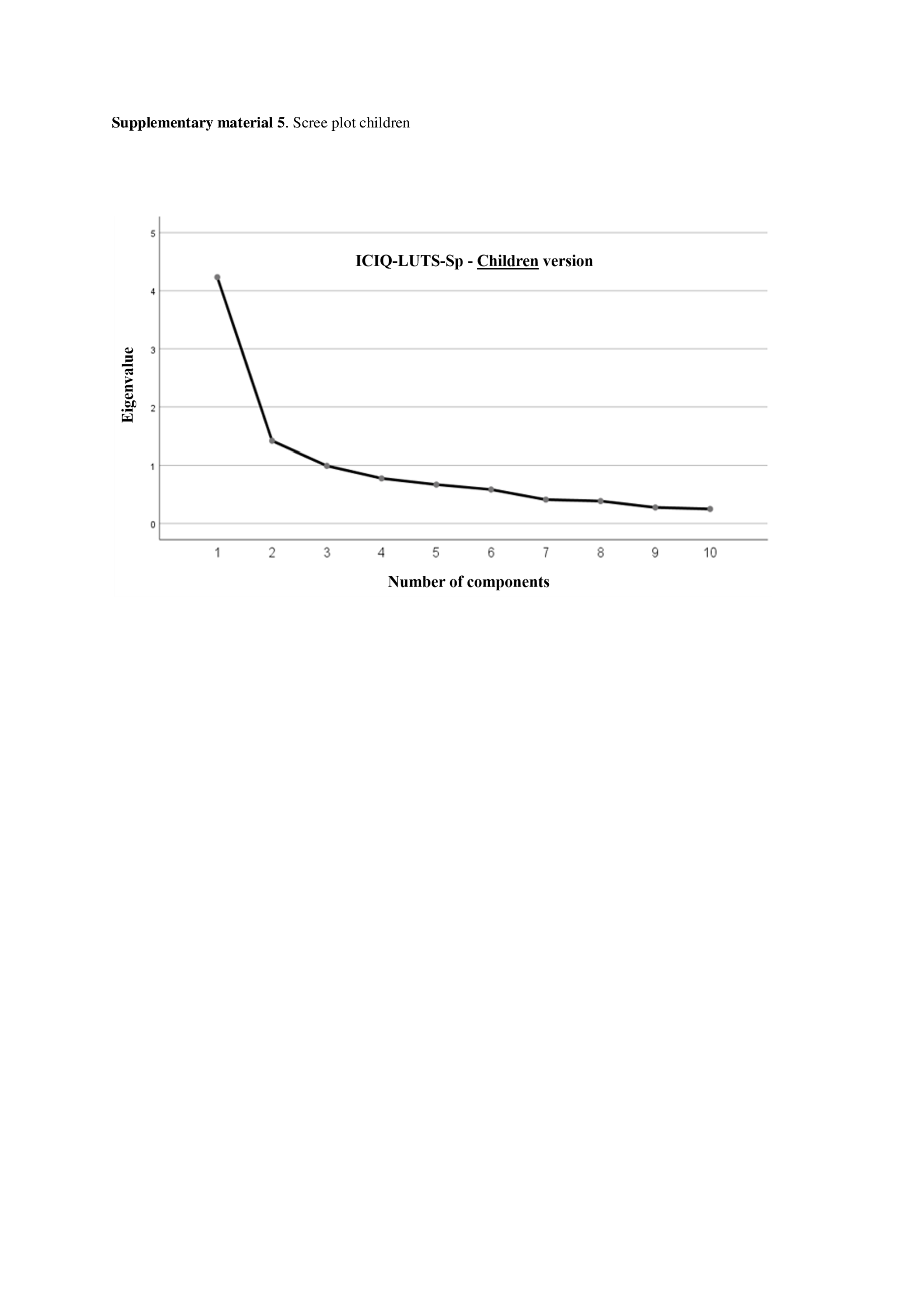

Supplement: Supplementary file 5 — Supplementary file5 Scree plot children (TIFF 30170 KB) [file 431_2023_4823_MOESM5_ESM.tiff]

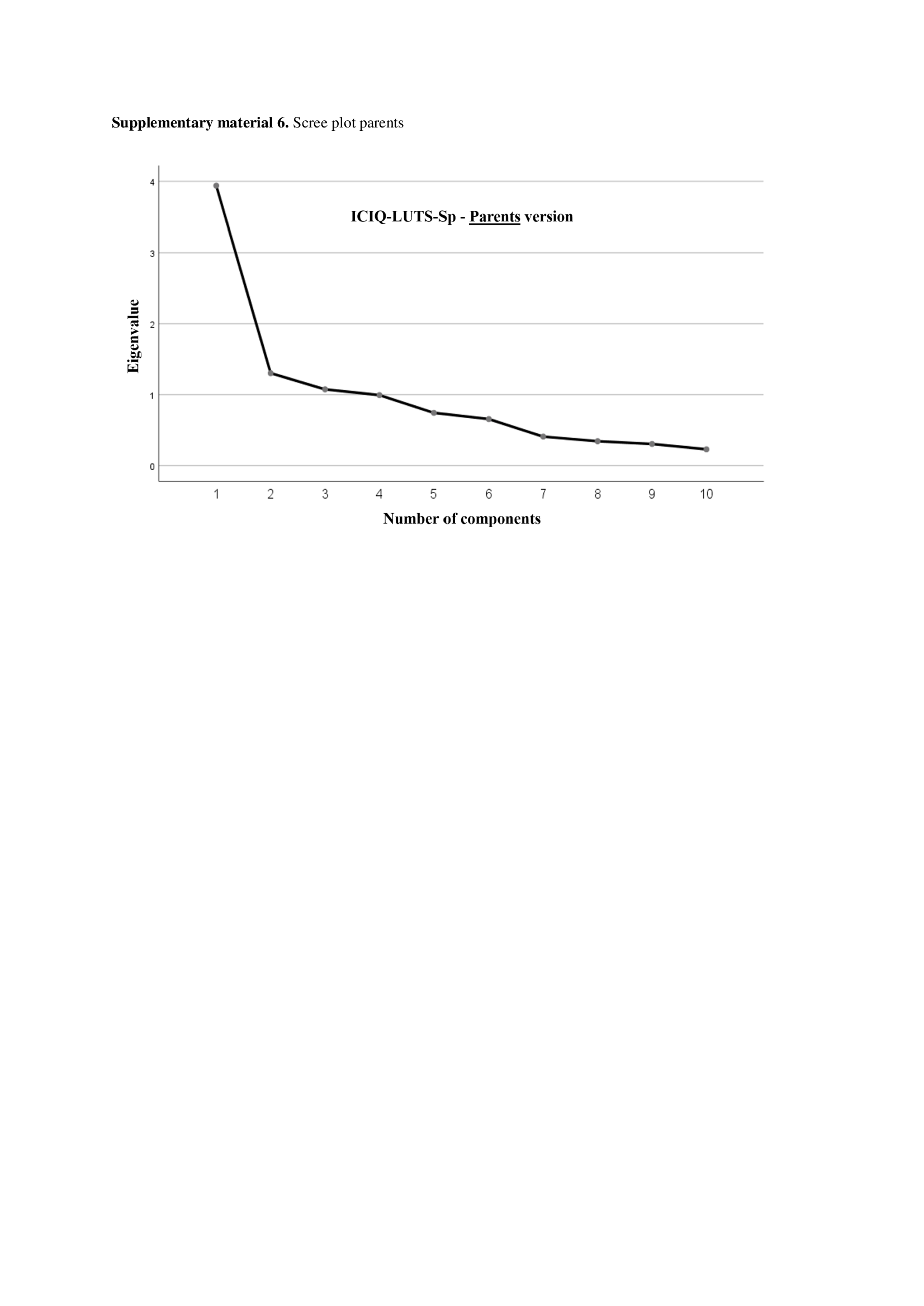

Supplement: Supplementary file 6 — Supplementary file6 Scree plot parents (TIFF 102 KB) [file 431_2023_4823_MOESM6_ESM.tiff]
